# Epidermal inclusion cyst as a rare complication of neonatal male circumcision: a case report

**DOI:** 10.4076/1752-1947-3-7321

**Published:** 2009-07-14

**Authors:** Linus Ikechukwu Okeke

**Affiliations:** 1 Department of Surgery, College of Medicine, University of Ibadan, University College Hospital, PMB 5116, Ibadan, Nigeria

## Abstract

**Introduction:**

Ibadan, Nigeria, has a very high rate of complications of male circumcision. In a previous survey, redundant or excessive loss of foreskin, skin bridges and injury to the glans penis were the major types of complications identified. Epidermal inclusion cyst complicating neonatal male circumcision appears to be extremely rare, and an extensive search of all databases revealed no reports in the recent literature.

**Case presentation:**

In 1992, a 10-year-old boy was seen at the urology outpatients clinic presenting with a globular swelling in the penile skin located at the ventral surface proximal to the coronal sulcus. The histology of the excised mass revealed an epidermal inclusion cyst. Since then, he has remained healthy.

**Conclusions:**

Epidermal inclusion cyst complicating male neonatal circumcision is extremely rare. The diagnosis is easy and a simple total excision is curative.

## Introduction

Neonatal circumcision is a common practice in Nigeria [1]. However, unlike other parts of the world where the incidence of complications arising from this procedure ranges between 0.19% and 3.1% [2,3], Nigeria has a rather high complication rate of 20.2% [1]. A search of all databases failed to return any reports of epidermal inclusion cyst as a complication of neonatal male circumcision; this rare event is thus reported here.

## Case presentation

**Figure 1:**
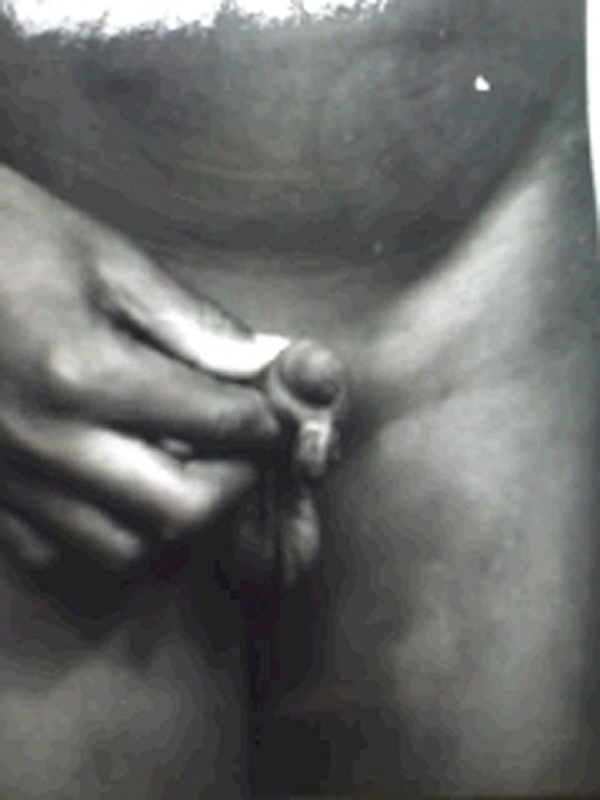
**Swelling located at the ventral surface of the penis, just proximal to the coronal sulcus; the circumcision scar runs over the swelling**.

A 10-year-old boy presented at the urology outpatients clinic in December 1992 with a 9-year history of a penile swelling. The swelling was located at the ventral aspect of the penis along the circumcision scar (Figure 1). It was painless and had been growing larger since it was first noticed despite the application of traditional remedies. There was no history of penile trauma apart from the circumcision, which had been performed at the age of 2 weeks. He had no associated urinary symptoms. On examination, the swelling was located at the ventral surface of the penis, just proximal to the coronal sulcus. It was globular in shape, measuring about 1 cm by 0.4 cm and had the circumcision scar running over it. The skin covering the swelling was otherwise normal. The swelling was firm, non-tender and was tethered to the circumcision scar. There were no other similar swellings in any other part of the body. It was excised under general anaesthesia and the histology was reported as an 'epidermal inclusion cyst'. Since then, the patient has remained well.

## Discussion

In a systematic review of the prevalence of complications of circumcision in anglophone Africa, Muula *et al.*[4] did not encounter any incidences of epidermal inclusion cyst. Understandably, a long period of follow-up is required before this complication can manifest itself. A search of all databases using the search terms 'epidermal inclusion cysts complicating male circumcision', 'epidermal inclusion cysts as a complication of neonatal circumcision', 'complications of male circumcision' and 'epidermoid inclusion cysts complicating neonatal male circumcision' failed to return any reports. Epidermal inclusion cysts would therefore appear to be an extremely rare complication of neonatal male circumcision.

The term 'epidermal inclusion cyst' has been used interchangeably with 'epidermal cyst' and 'epidermoid cyst' in the medical literature. These cysts result from the proliferation of epidermal cells within a circumscribed space of the dermis and appear as firm, round, mobile, subcutaneous nodules of variable size. A central pore or punctum that may tether the cyst to the overlying epidermis is an inconsistent finding.

This was not present in this patient but his cyst was tethered to his circumcision scar. These cysts can occur in various parts of the body but are found most commonly in the vulva, especially in populations where female circumcision is practised [5,6]. In addition to arising from surgical implantation of epidermal tissue, as in this patient, these cysts may also arise from the sequestration of epidermal rests during embryonic life, occlusion of the pilosebaceous unit or traumatic implantation of epithelial elements. The patient had no other lumps in other parts of his body thus excluding certain hereditary syndromes such as Gardner syndrome. A simple total excision was curative and he has remained healthy with no recurrence for 15 years.

## Conclusions

Epidermal inclusion cyst complicating male neonatal circumcision is extremely rare. The diagnosis is easy and a simple total excision is curative.

## Consent

Written informed consent was obtained from the patient's next-of-kin for publication of this case report and any accompanying images. A copy of the written consent is available for review by the Editor-in-Chief of this journal.

## Competing interests

The author declares that he has no competing interests.
